# Structure of trophic and mutualistic networks across broad environmental gradients

**DOI:** 10.1002/ece3.1371

**Published:** 2014-12-23

**Authors:** Ellen A R Welti, Anthony Joern

**Affiliations:** Division of Biology, Kansas State University116 Ackert Hall, Manhattan, Kansas, 66506-4901

**Keywords:** Community stability, complexity, ecological gradients, ecological networks, mutualism, trophic interactions

## Abstract

This study aims to understand how inherent ecological network structures of nestedness and modularity vary over large geographic scales with implications for community stability. Bipartite networks from previous research from 68 locations globally were analyzed. Using a meta-analysis approach, we examine relationships between the structure of 22 trophic and 46 mutualistic bipartite networks in response to extensive gradients of temperature and precipitation. Network structures varied significantly across temperature gradients. Trophic networks showed decreasing modularity with increasing variation in temperature within years. Nestedness of mutualistic networks decreased with increasing temperature variability between years. Mean annual precipitation and variability of precipitation were not found to have significant influence on the structure of either trophic or mutualistic networks. By examining changes in ecological networks across large-scale abiotic gradients, this study identifies temperature variability as a potential environmental mediator of community stability. Understanding these relationships contributes to our ability to predict responses of biodiversity to climate change at the community level.

## Introduction

**Figure 1:**
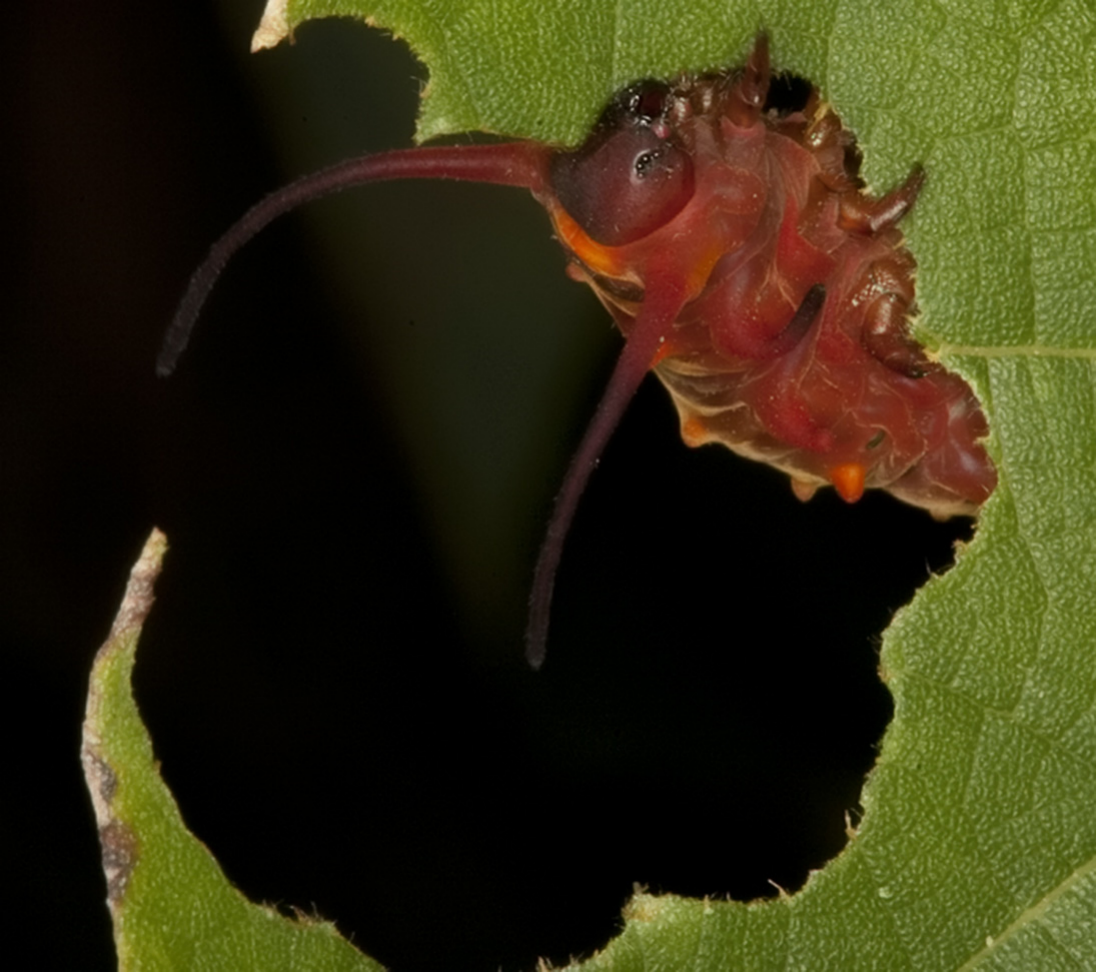
A pipevine-swallowtail caterpillar (*Battus philenor*) feeds on a host plant (*Aristolochia* spp.). Interactions between insect herbivores and their host plants at the community level can have nonrandom structural properties which vary across environmental gradients.

Understanding changes in community dynamics along major environmental gradients is a major goal of community ecology. Substantial ecologically relevant gradients abound, including abiotic ones such as precipitation, temperature, or salinity gradients (Crain et al. 2008; Kaspari et al. 2000), changes in biotic environments resulting from variable primary productivity, habitat structure, and gradients associated with competition or predation risk (Ripley and Simovich 2009; Ricklefs 2004). Changing species diversity along productivity gradients (Tilman et al. 2012), the relationship between food web complexity and stability (Krause et al. 2003), variable abiotic conditions and the likelihood of trophic cascades (Laws and Joern 2013), or changes with niche metrics such as diet breadth or overall community stability in response to species diversity (Haddad et al. 2011; Pianka 1973, 1966a,b) are all examples of long-standing interest in this context. Despite much success in identifying single species responses and ecosystem-level responses to underlying gradients, a great need remains to understand how networks of coexisting species respond (Bascompte 2010), or how the observed network structure reflects species diversity. For example, ecological gradients may affect community dynamics through limiting species richness (Dyer et al. 2007); alternatively, environmental conditions may directly influence species interactions and thus community stability and species richness. Here, we assess changes in communities over gradients of precipitation and temperature using an ecological network framework (Fig. 1).

In studies of ecological networks, ecologists focus on the role played by species linkages to assess the overall functional stability or persistence of a network (Bascompte 2010), or they predict likely changes in community persistence when components are removed (Pocock et al. 2012). The ecological network approach emphasizes species interactions and internal architecture of linkages in communities as important factors affecting species persistence across changing environmental conditions (Pearson and Dawson 2003). The approach benefits from methodological contributions across many disciplines, including physics and sociology (Bascompte 2009).

In an ecological network framework, communities are represented as adjacency matrices with axes composed of plants (*i*) and consumer (*j*) species with the goal of assessing how network structural characteristics such as nestedness and modularity covary with community traits and stability (Montoya et al. 2006). Theoretical studies have linked nestedness to the stability of mutualistic networks (Thébault and Fontaine 2010). In nested networks, specialist species interact primarily with generalist species, which tend to be the more persistent and stable members of the community. A nested structure may allow rare specialist species to persist because the limited numbers of species with which they interact are maintained by generalist species (Jordano 1987). Recent work has found nestedness to be less important for individual species persistence than the simpler metric of number of mutualistic partners (James et al. 2012). However, nestedness may stabilize mutualistic networks at the community level by increasing the number of mutualistic animal partners shared by plants and the number of plants shared by animals, therefore decreasing competition between plants and between animals (Bastolla et al. 2009).

Modularity has been linked theoretically to stability in trophic networks (Thébault and Fontaine 2010), although empirical evidence is limited (Krause et al. 2003). Modularity is hypothesized to increase ecological network stability by limiting the spread of a perturbation to the confines of the compartment of the perturbation's origin (Montoya et al. 2006). Larger mutualistic networks have also been shown to be significantly modular (Olesen et al. 2007) although the significance of modularity in mutualistic networks is understudied.

By comparing network types, we can identify differences in community dynamics due to mutualistic versus antagonistic interactions (Thébault and Fontaine 2010). Here, we evaluate and compare trophic (insect herbivore–plant) and mutualistic (pollinator–plant and seed-disperser plant) networks, most of which are insect–plant interaction networks. Because insects and plants comprise disproportionately large groups of global biodiversity, studies of their interactions are well represented in the literature and make insect–plant interaction networks a suitable choice for comparative analysis.

Network structure is often highly correlated with species richness (Olesen et al. 2007; Fonseca et al. 2005; Jordano 1987). Prior studies predict that modularity increases with species richness (Olesen et al. 2007), whereas nestedness decreases with species richness (James et al. 2012; Fonseca et al. 2005). We include species richness in our models predicting network structure to account for its contribution while we consider the effects of other factors, and consider the model with species richness as the only predictor variable to be our null model.

Annual cumulative temperature is an indicator of growing season length, a limiting factor for many plant and animal communities. A known source of nestedness in plant–pollinator networks is the preferential association of incoming pollinators with the most highly linked plants in a network (Olesen et al. 2008). We hypothesize that there will be positive relationship between annual cumulative temperature and nestedness of mutualistic networks, which is the result of a longer growing season, allowing for further development of such linkages and therefore increasing nestedness. Variability in growing season length should disrupt this assembly process, potentially resulting in more fragmentation and network modularity. In trophic networks, we hypothesize that herbivores entering the community do not preferentially eat plants with the most links but instead are limited by nutritional niche space (Behmer and Joern 2008; Guimerá et al. 2010), phylogeny affecting host plant use (Rezende et al. 2009), plant defensive compounds and micronutrients (Rosenthal and Berenbaum 1992; Becerra 2007; Joern et al. 2012). As growing season length increases, so does the number of interacting and coevolving insects and plants. Variability in growing season length should reduce the size and number of these modules and force species to become more generalist in their resource use to survive, increasing network nestedness. If modularity is associated with stability in trophic networks and nestedness with the stability of mutualistic networks as predicted theoretically (Thébault and Fontaine 2010), ecological communities existing in areas with longer and less variable growing seasons are predicted to be more stable than those in areas with shorter and more variable growing seasons.

Likewise, precipitation should have a positive relationship with total resource availability for insects including increases in plant biomass for herbivorous insects and potentially flowering plant diversity for pollinating insects. We predict nestedness of mutualistic networks, and modularity of trophic networks will increase with mean annual precipitation and decrease with precipitation variability.

Because changes in network structures are putatively associated with community stability (Thébault and Fontaine 2010), understanding the influence of environmental conditions on network structure should provide insight into causes of stability and fragility in ecological communities as conditions change in either time or space. As such, knowing the relationships between ecological gradients and ecological network structures could help to predict persistence of species facing global climate change.

## Materials and Methods

### Datasets

Bipartite mutualistic and trophic networks were collected from published studies (Dyer et al. 2007; Rezende et al. 2007; Joern 1983, for full list of network sources please see Appendix S1 in Supporting Information). Mutualistic networks included 25 plant–pollinator and 21 seed-disperser networks. Trophic networks included 22 plant–insect herbivore networks.

### Environmental variables

The geographic location of each network was plotted using Google Earth, and all points were converted into a kmz document. This document was overlaid on NASA Earth Observatory (NEO) (EOS Project Science Office 2013) cumulative monthly data maps of precipitation and temperature for all months from 2001 to 2012. These years were selected for analyses because they are years for which NEO data were available for all months. Point values were extracted in ArcGIS 10.1 (ESRI 2012) for each map at each network location. We used these data to calculate 12-year averages, coefficient of variation (CV) among years, and CV within years for precipitation and temperature for each network site. Precipitation maps provided data only for locations between 35° N and S latitude. Because of this constraint, networks from the source studies located outside of this range were not included in the analysis.

### Network structural properties

We analyzed nestedness and modularity of all networks using standard metrics. Modularity is the tendency for organisms to interact in subgroups (called modules) and not to interact with organisms outside of their module. Modularity calculations were made using the Newman and Girvan (2004) algorithm in the software BIPMOD (Thébault 2013). Nestedness is a measure of the degree to which specialist species’ interactions are a subset of generalist species’ interactions (Bascompte 2010). The NODF (nested metric based on overlap and decreasing fill) metric for nestedness was used in this study. NODF is preferred to alternate metrics based on deviations from a maximum nestedness value, which have been shown to inflate the type I error (Almeida-Neto et al. 2008). NODF was calculated using the software ANINHADO ver. 3.0.3 3 (Guimarães and Guimarães 2006).

### Statistics

Relationships between environmental variables and their variability and network structures were analyzed following Akaike's information criterion (ΔAIC_c_) (Burnham and Anderson 2002). Global models for the response variables of nestedness and modularity were analyzed using combinations of predictor variables including species richness, mean annual cumulative temperature, the coefficient of variation (CV) of temperature between years, the CV of temperature within years, mean annual cumulative precipitation, the coefficient of variation (CV) of precipitation between years, and the CV of precipitation within years. The global model and all reduced additive models from the global model were fitted using the *dredge* function in the MuMIn package (Barton 2012) in R ver. 3.0.2 (R Development Core Team 2014). The model with only species richness as a predictor variable was considered the null model; if this null model had a ΔAIC_c_ < 2, other models were considered irrelevant. Otherwise, models with ΔAIC_c_ < 2 were considered equally parsimonious. The relative importance values (RIV) for each predictor variable, computed as the sum of Akaike weights (*w*_*i*_), were also calculated. Because nestedness and species richness were log-normally distributed in previous studies (Dalsgaard et al. 2013; Fonseca et al. 2005; Bengtsson 1994), nestedness and species richness were log_10_-transformed for all analyses. Variability of environmental variables was measured as the coefficient of variation (CV) between years (CV of annual cumulative sums) and within years (CV of monthly sums).

## Results

### Nestedness of mutualistic networks

**Figure 2:**
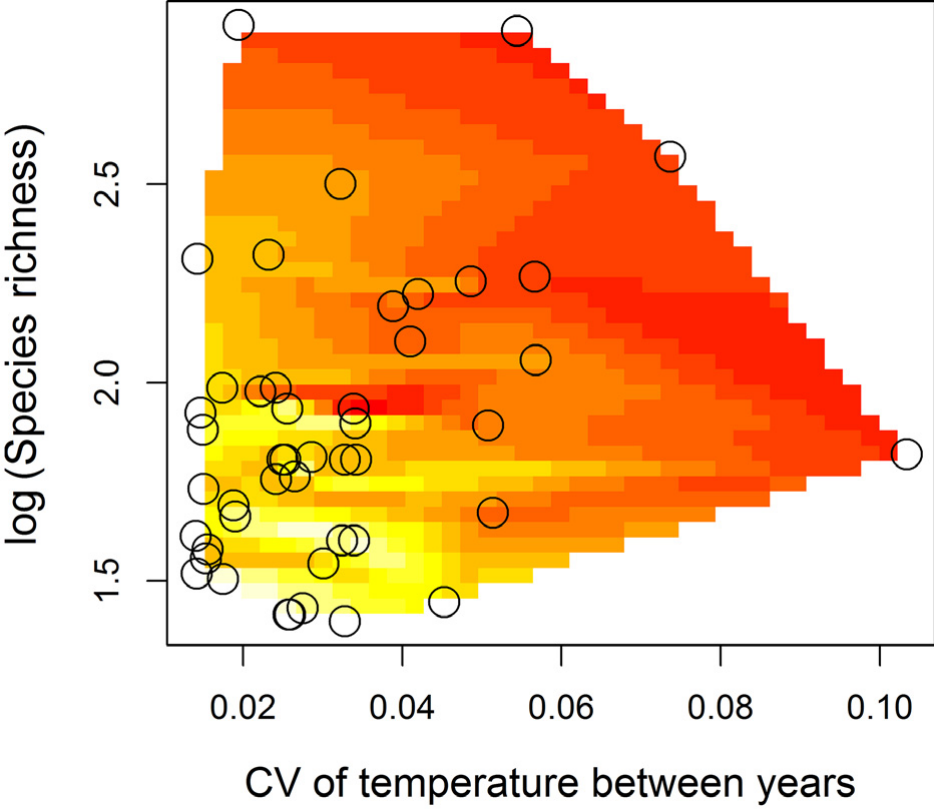
Contour plot for the relationships between the coefficient of variation of temperature between years, species richness, and nestedness of mutualistic networks. Color is used to represent nestedness. Lighter colors (yellow) indicate high nestedness values while darker colors (red) indicate low nestedness values.

The likelihood of 127 competing models comprising the global models and all reduced forms of the global model was assessed using AIC_c_ analysis. The predictor variables in the global model and their abbreviations are listed in Table1. Only one model explaining variation in the nestedness of mutualistic networks of the 127 competing models had a ΔAIC_c_ <2, indicating it is the best fitting model. This model included species richness and the CV of temperature between years as the only variables explaining variation in the nestedness of mutualistic networks (Table2, Part A). The CV of temperature between years also had a high RIV, indicating it is an important predictor of the nestedness of mutualistic networks (Table3, Part A). Nestedness of mutualistic networks decreased with both species richness and the CV of temperature between years (Fig.2).

**Table 1:** Variables included in global model for analyses of relationships between network structures (nestedness and modularity) and environmental variables. Species richness was included to account for network structure variation due to network size

Abbreviation	Variable
spp_richness	Total number of species in the network (plants + animals)
Precip	12 year average mean annual precipitation (mm)
CVprecipBTWyrs	Coefficient of variation of mean annual precipitation between years
CVprecipW/INyrs	Coefficient of variation of mean annual precipitation within years
Temp	12 year average mean cumulative annual temperature (°C)
CVtempBTWyrs	Coefficient of variation of mean cumulative temperature between years
CVtempW/INyrs	Coefficient of variation of mean cumulative temperature within years

**Table 2:** AIC_c_ statistics for models for network nestedness and modularity for mutualistic and trophic networks. *AIC*_*c*_ = AIC corrected for small sample size, *LL* = log likelihood, *df* = degrees of freedom, *R*^2^ = adjusted regression coefficient, *P* = model *P*-value, ΔAIC_*c*_ = difference between the top model and given model AIC_c_, *w*_*i*_ = model weight. Only models with ΔAIC_*c*_ < 2 are shown for each network structure/network type comparison. If the model with only species richness was included as a model with ΔAIC_*c*_ < 2, the accompanying models were not considered statistically meaningful

Model variables	AIC_c_	LL	df	*R* ^2^	*P*	ΔAIC_c_	*w*_*i*_
A. Nestedness of mutualistic networks							
Spp_richness, CVtempBTWyrs	8.02	0.5	4	0.44	2E-06	0	0.33
B. Modularity of mutualistic networks							
Spp_richness, CVtempBTWyrs	−66.37	37.7	4	0.25	7E-04	0	0.14
Spp_richness	−65.76	36.2	3	0.22	6E-04	0.62	0.10
Spp_richness, temp	−65.52	37.2	4	0.24	0.001	0.85	0.09
Spp_richness, CVtempW/INyrs	−65.32	37.1	4	0.24	0.001	1.06	0.08
C. Nestedness of trophic networks							
Spp_richness, CVtempBTWyrs	15.07	−2.4	4	0.73	1E-06	0	0.13
Spp_richness, CVtempW/INyrs	15.32	−2.5	4	0.73	2E-06	0.25	0.11
Spp_richness	16.04	−4.4	3	0.70	9E-07	0.97	0.08
Spp_richness, temp	16.08	−2.9	4	0.72	2E-06	1.01	0.08
Spp_richness, CVprecipW/INyrs	16.29	−3.0	4	0.72	2E-06	1.22	0.07
Spp_richness, CVprecipBTWyrs, CVtempBTWyrs	16.69	−1.5	5	0.74	4E-06	1.62	0.06
Spp_richness, CVprecipBTWyrs, CVprecipW/INyrs	16.88	−1.6	5	0.74	5E-06	1.81	0.05
D. Modularity of trophic networks							
Spp_richness, CVtempW/INyrs	−52.53	31.4	4	0.89	3E-10	0	0.30
Spp_richness, CVprecipW/INyrs, CVtempW/INyrs	−50.83	32.3	5	0.89	2E-09	1.70	0.13
Spp_richness, CVprecipW/INyrs, temp, CVtempW/INyrs	−50.55	34.1	6	0.90	4E-09	1.98	0.11

**Table 3:** Relative importance values of predictor variables for all models

Spp_richness	Precip	CVprecipBTWyrs	CVprecipW/INyrs	Temp	CVtemp BTWyrs	CVtemp W/INyrs
A. Nestedness of mutualistic networks						
1	0.23	0.22	0.24	0.28	0.74	0.31
B. Modularity of mutualistic networks						
0.97	0.31	0.29	0.23	0.33	0.43	0.3
C. Nestedness of trophic networks						
0.99	0.2	0.34	0.37	0.25	0.34	0.32
D. Modularity of trophic networks						
1	0.16	0.17	0.36	0.34	0.29	0.76

### Modularity of mutualistic networks

Four top models with ΔAIC_c_ < 2 resulted from the AIC_c_ analysis of the modularity of mutualistic networks. These models included a model with mean annual cumulative temperature, a model including the coefficient of variation (CV) of temperature between years, and a model including the CV of temperature within years. However, the null model (model including only species richness as a predictor of modularity) was also included in the top models (Table2, Part B); therefore, there is no strong evidence for a correlative relationship between environmental variables and the modularity of mutualistic networks. Likewise, the RIVs of environmental variables as predictors of the modularity of mutualistic networks are uniformly low (Table3, Part B).

### Nestedness of trophic networks

There were seven top models with ΔAIC_c_ < 2 predicting nestedness of trophic networks. Mean annual cumulative temperature, the coefficient of variation (CV) of temperature between years, the CV of temperature within years, the coefficient of variation (CV) of precipitation between years, and the CV of precipitation within years were all included in the top models. However, the null model including only species richness as a predictor of the nestedness of trophic networks was also included in the top models (Table2, Part C), so the additional models are considered ecologically irrelevant. The RIVs of environmental variables were also low, indicating a lack of evidence for the influence of environmental variables on the nestedness of trophic networks (Table3, Part C).

### Modularity of trophic networks

**Figure 3:**
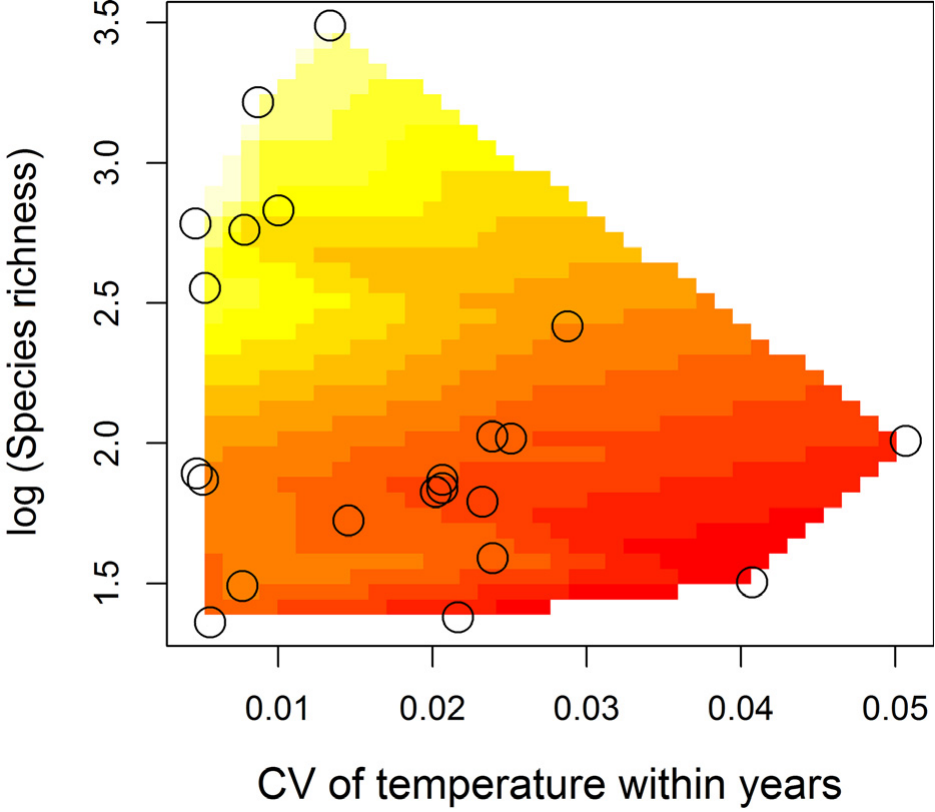
Contour plot for the relationships between the coefficient of variation of temperature within years, species richness, and modularity of trophic networks. Color is used to depict modularity. Lighter colors (yellow) indicate high modularity, and darker colors (red) indicate low modularity values.

Three top models with ΔAIC_c_ < 2 resulted from the AIC_c_ analysis of the modularity of trophic networks. Besides species richness, the CV of temperature within years explained a significant portion of the variation in the modularity of trophic networks and was included in all three top models (Table2, Part D). The CV of precipitation within years and cumulative mean annual temperature were also included as predictor variables in plausible models, but the RIV of the CV of temperature within years is more than twice as high as all other environmental variables (Table3, Part D). Modularity of trophic networks increased with species richness and decreased with the CV of temperature within years (Fig. 3).

## Discussion

Ecological networks have inherent structure (Bascompte 2010). Although many possible drivers of ecological network structure have been proposed, sources of variation in network structure remain elusive (Thébault and Fontaine 2010). Network nestedness has been hypothesized to arise from network size (Bastolla et al. 2009; James et al. 2012), interaction strength (Okuyama and Holland 2008; Suweis et al. 2013), interaction switches (Zhang et al. 2011), extinction events (Thébault and Fontaine 2010), and phylogenic relatedness (Rezende et al. 2007, 2009). Modularity has been hypothesized to be linked to network size (Olesen et al. 2007), habitat structure (Pimm and Lawton 1980), niche space (Guimerá et al. 2010), trait matching (Joppa and Williams 2013), phylogeny (Rezende et al. 2007), and rate of temperature change (Dalsgaard et al. 2013). In sum, much theoretical modeling of ecological networks predicts that network structure arises from combined contributions from multiple sources. Here, we assess how environmental conditions may influence network structure directly, or they may work in tandem with other sources of network structure. Moreover, predicted relationships between network structure and species richness often do not match the available empirical data (James et al. 2012), suggesting that further comparative synthesis of current models and empirical testing of hypothesized drivers of network structures is needed. This study investigates the hypothesis that broad-scale environmental conditions are drivers of network structure and explain much variation in community network structure observed at a geographic scale.

### Network structure and environmental variables

We document an inherent difference of network structure between trophic and mutualistic networks in response to temperature variation over broad geographic gradients. In mutualistic networks, increases in temperature variation between years corresponded to increases in nestedness. In trophic networks, as temperature variation within years increased, modularity decreased. The effect of temperature variation on network structure was strong even when the effect of species richness was included in the models. These results are consistent with our hypothesis that temperature variability should decrease nestedness in mutualistic networks and modularity in trophic networks. Neither mutualistic nor trophic network structures were significantly correlated with precipitation variables. This suggests that precipitation and variability of precipitation are not primary drivers of network structure, although they may influence network structure indirectly, such as through relationships between species richness and net primary productivity.

To our knowledge, this is the first study to test the potential of broad-scale temperature and precipitation gradients to predict both trophic and mutualistic network structure on a global scale. This extends greatly projections of previous case studies that found correlations between structure and environmental properties. Modularity decreased with latitude, and contrary to our results, precipitation was strongly correlated with nestedness and modularity in a comprehensive analysis of 54 mutualistic networks (Trøjelsgaard and Olesen 2013). In a comparative study of stream food webs, Thompson and Townsend (2005) linked network connectance to fine particulate matter. Soil fertility across a forest-brush gradient in southern Brazil accompanied network connectance through controlling species richness (Fonseca et al. 2005). Phenophase length was correlated with number of links per species in a study of temporal changes in a Greenland plant–pollinator network (Olesen et al. 2008). Such examples suggest that large-scale environmental factors can influence network assembly.

Our results also extend inferences of a small number of large-scale studies that examined potential effects of environmental drivers on network structure. Net primary productivity explained 17% of the variance in 14 multitrophic food webs (Vermaat et al. 2009). In plant–pollinator networks, the number of interactions per plant species decreased on islands compared to mainland, and connectance of residuals increased from highland to lowland (Olesen and Jordano 2002). A recent analysis of the effects of global climate change on plant–pollinator networks showed reduced modularity in pollination networks when associated with high rate of climate change in the Quaternary (last 2.6 million year) (Dalsgaard et al. 2013). In light of these studies, there is definitely reason to expect changes in network structure along large-scale environmental gradients.

### Network structure and species richness

While species richness can be significantly correlated with network modularity (Jordano 1987; Olesen and Pedro 2002) and nestedness (James et al. 2012; Dalsgaard et al. 2013), the causal significance of species richness to network structure remains a long-standing, unresolved question. Some studies suggest mutualistic networks increase in nestedness as they increase in size (Okuyama and Holland 2008; Suweis et al. 2013). In agreement with our results, recent meta-analyses of mutualistic networks found nestedness decreased significantly with increased species richness (James et al. 2012; Dalsgaard et al. 2013). Modularity has been shown to increase with species richness (Olesen et al. 2007; Dalsgaard et al. 2013), a result duplicated in our analysis.

One hypothesis for explaining the relationship between network structure and species richness is that the size of the network constrains network structure (Fontaine 2013). For example, the nature of a smaller network requires it to have high connectivity for all network members to be included (Fonseca et al. 2005). Likewise, modularity is not expected in small networks because the limited number of interactions is not sufficient to allow partitioning into modules. Another hypothesis suggests that increases in network size are driven by network structure (Okuyama and Holland 2008; Bascompte 2009; Suweis et al. 2013). Because the relationship between species richness and network structure did not differ regardless of network type, our results do not refute either of these hypotheses. Therefore, the effect of species richness must be accounted for when testing for the influence of other variables on network structure (Bengtsson 1994; Fonseca et al. 2005).

### Potential sources of error

As for most community-scale studies, results of this study may be biased by incomplete data from missing species. Network structure reflects species presence and the organization of species interactions. If species or interactions are not included in the network, the calculated network structure is incomplete, potentially altering conclusions. Because most trophic networks used in this study are based on studies with multiple years of sampling, we feel they are reliable. The mutualistic networks are more variable in degree of sampling completeness, but represent the best available at the geographic scale of this study.

While ecological networks go beyond species richness in describing community structure by including species interactions, bipartite networks such as those used here clearly do not fully capture the full complexity of ecological communities. Incorporating weighted matrices, where interaction strength is measured, is a logical next step in ecological network studies. However, the sampling effort necessary to accurately identify all species in a community, the species with whom they interact, and the weight of interactions remains a great challenge.

### Importance and future directions

Our study seeks to understand the long-standing question of why and how environmental gradients influence species richness and community dynamics. Documenting only changes in species richness is not sufficient for determining changes in ecosystem functioning and services. For example, in one well-sampled and taxonomically well-resolved study of bees, important insect pollinators, the community decreased in diversity by 50% in the last 120 years. However, the number of interactions between the bee species and the angiosperm species in the system decreased at a greater rate of 76% over the same time frame (Burkle et al. 2013). Interaction number in plant–pollinator communities is more important than number of pollinator species for the desired ecosystem service of pollination.

May (1972) noted that stability is not an inherent property of complexity in random communities, although modularity was proposed as the missing structure that stabilizes such food webs (Lawlor 1978). Ecological modeling has since provided much additional support for the hypothesis that nonrandom network structures such as modularity and nestedness increase the stability of ecological networks (Okuyama and Holland 2008; Thébault and Fontaine 2010). Ecological models have been less succinct in predicting the cause(s) of network structure, as different models demonstrated that multiple factors may be drivers of the same network structure. We show that network type and temperature variables can influence network structure over broad environmental gradients, to then be refined by local conditions and interactions.

Future research must assess whether environmental conditions such as those we evaluated actually drive network structure or are correlated for other reasons (i.e., correlation does not always translate into causation). If environmental properties drive network structure, understanding the timescale over which change in environment effects change in network structure and the robustness of networks to changes becomes critical. Due to the difficulty of experimental tests of changes in network structure, especially under field conditions, empirical evidence for direct causes of observed structure along large environmental gradients remains elusive. However, understanding network structure is critical as it offers potential insight into community resilience and stability, leading to better predictions of the impacts of changing environmental conditions at the global level on ecological communities and ecosystem function in this period of unprecedented change.

## Supporting Information

**Appendix S1.** Data Source References for Structure of trophic and mutualistic networks across broad environmental gradients and other trophic network sources.
