# Supplementary material for: Structure of trophic and mutualistic networks across broad environmental gradients
Source: Ecol Evol. 2014 Dec 23;5(2):326–34. doi: 10.1002/ece3.1371 (PMC4314265; doi:10.1002/ece3.1371)
Supplement: Supplementary file 1 [file ece30005-0326-sd1.docx]

**Supporting Information, Appendix 1**

**Data Source References for**

**Structure of Trophic and Mutualistic Networks across Broad Environmental Gradients**

**Welti & Joern 2014**

**Mutualistic networks** (from Rezende, E., Lavabre, J., Guimarães, P., Jordano, P., Bascompte, J. & Guimarães, P. (2007) Non-random coextinctions in phylogenetically structured mutualistic networks. Nature, 448, 925-8.)**:**

Arroyo, M.T.K., Primack, R. & Armesto, J.J. (1982) Community studies in pollination ecology in the high temperate Andes of central Chile. I. Pollination mechanisms and altitudinal variation. American Journal of Botany. 69, 82-97.

Baird, J.W. (1980) The selection and use of fruit by birds in an eastern forest. Wilson Bulletin, 92, 63-73.

Barrett, S. C. H., & Helenurm, K. (1987) The Reproductive-Biology of Boreal Forest Herbs.1. Breeding Systems and Pollination. Canadian Journal of Botany, 65, 2036-2046.

Beehler, B. (1983) Frugivory and polygamy in birds of paradise. Auk, 100: 1-12.

Bezerra, E.L.S, Machado, I. C. & Mello, M.A.R. (2009). Pollination networks of oil-flowers: a tiny world within the smallest of all worlds. Journal of Animal Ecology, 78, 1096-1101.

Carlo et al. (2003) Avian fruit preferences across a Puerto Rican forested landscape: pattern consistency and implications for seed removal. Oecologia 134: 119-131

Clements, R. E., & Long, F.L. (1923) Experimental pollination. An outline of the ecology of flowers and insects. Washington, D.C., USA, Carnegie Institute of Washington.

Crome, F.H.J. 1975. The ecology of fruit pigeons in tropical Northern Queensland. Australian Journal of Wildlife Research, 2: 155-185.

Dicks, L.V., Corbet, S.A. & Pywell, R.F. (2002) Compartmentalization in plant–insect flower visitor webs. Journal of Animal Ecology, 71, 32–43.

Dupont YL, Hansen DM and Olesen JM (2003) Structure of a plant-flower-visitor network in the high-altitude sub-alpine desert of Tenerife, Canary Islands. Ecography 26:301-310.

Eskildsen et al. unpubl.

Frost, P.G.H. (1980). Fruit-frugivore interactions in a South African coastal dune forest. Pages 1179-1184 in: R. Noring (ed.). Acta XVII Congresus Internationalis Ornithologici, Deutsches Ornithologische Gessenshaft, Berlin.

Galetti, M., Pizo, M.A. 1996. Fruit eating birds in a forest fragment in southeastern Brazil. Ararajuba, Revista Brasileira de Ornitologia, 4: 71-79.

Hammann, A. & Curio, B. (1999) Interactions among frugivores and fleshy fruit trees in a Philippine submontane rainforest. Conservation Biology 13: 766-773.

Herrera, J. (1988) Pollination relatioships in southern spanish mediterranean shrublands. Journal of Ecology 76: 274-287.

Inouye, D. W., & Pyke, G.H. (1988) Pollination biology in the Snowy Mountains of Australia: comparisons with montane Colorado, USA. Australian Journal of Ecology 13, 191-210.

Jordano P. (1985) El ciclo anual de los paseriformes frugívoros en el matorral mediterráneo del sur de España: importancia de su invernada y variaciones interanuales. Ardeola, 32, 69-94.

Kantak, G.E. 1979. Observations on some fruit-eating birds in Mexico. Auk, 96: 183-186.

Kato, M., Kakutani, T., Inoue, T. & Itino, T. (1990) Insect-flower relationship in the primary beech forest of Ashu, Kyoto: An overview of the flowering phenology and the seasonal pattern of insect visits. Contributions to the Biology Laboratory, Kyoto, University, 27, 309-375.

Lambert F. (1989) Fig-eating by birds in a Malaysian lowland rain forest. Journal of Tropical Ecology, 5, 401-412.

Mack, AL and Wright, DD. (1996) Notes on occurrence and feeding of birds at Crater Mountain Biological Research Station, Papua New Guinea. Emu 96: 89-101.

Medan, D., Montaldo, N.H., Devoto, M., Mantese, A., Vasellati, Y., & Bartoloni, N.H. (2002) Plant-pollinator relationships at two altitudes in the Andes of Mendoza, Argentina. Arctic and Antarctic Alpine Research, 34, 233-241.

Memmott J. (1999) The structure of a plant-pollinator food web. Ecology Letters, 2, 276-280.

Motten, A. F. (1982) Pollination Ecology of the Spring Wildflower Community in the Deciduous Forests of Piedmont North Carolina. Doctoral Dissertation thesis, Duke University, Duhram, North Carolina, USA; Motten, A. F. 1986. Pollination ecology of the spring wildflower community of a temperate deciduous forest. Ecological Monographs, 56, 21-42.

Noma, N. (1997) Annual fluctuations of sapfruits production and synchronization within and inter species in a warm temperate forest on Yakushima Island, Japan. Tropics, 6, 441-449.

Olesen unpubl.

Ollerton, J., Johnson, S.D., Cranmer, L. & Kellie, S. (2003) The pollination ecology of an assemblage of grassland asclepiads in South Africa. Annuals of Botany, 92, 807-834.

Percival, M. (1974). Floral ecology of coastal scrub in southeast Jamaica. Biotropica, 6, 104-129.

Petanidou, T. (1991). Pollination ecology in a phryganic ecosystem. Unp.PhD. Thesis, Aristotelian University, Thessaloniki.

Primack, R.B. (1983) Insect pollination in the New Zealand mountain flora. New Zealand Journal of Botany, 21, 317-333.

Ramirez, N. (1989). Biología de polinización en una comunidad arbustiva tropical de la alta Guyana Venezolana. Biotropica, 21, 319-330.

Ramirez, N. & Brito, Y. (1992). Pollination Biology in a Palm Swamp Community in the Venezuelan Central Plains. Botanical Journal of the Linnean Society, 110, 277-302.

Santos, G.M.M, Aguiar, C.M.L. & Mello, M.A.R. (2010). Flower-visiting guild associated with the Caatinga flora: trophic interaction networks formed by social bees and social wasps with plants. Apidologie, 41, 466-475.

Schemske, D. W., Willson, M. F., Melampy, M. N., Miller, F.J., Verner, L., Schemske, K.M. & Best, L. B. (1978) Flowering Ecology of Some Spring Woodland Herbs. Ecology, 59, 351-366.

Silva, W.R., P. De Marco, E. Hasui, and V.S.M. Gomes (2002) Patterns of fruit-frugivores interactions in two Atlantic Forest bird communities of South-eastern Brazil: implications for conservation. Pp. 423-435. In: D.J. Levey, W.R. Silva and M. Galetti (eds.) Seed dispersal and frugivory: ecology, evolution and conservation. Wallinford: CAB International.

Small, E. (1976) Insect pollinators of the Mer Bleue peat bog of Ottawa. Canadian Field Naturalist, 90, 22-28.

Smith-Ramírez, C., Martinez, P., Nuñez, M., González C. & Armesto J.J. (2005) Diversity, flower visitation frequency and generalism of pollinators in temperate rain forests of Chiloé Island, Chile. Botanical Journal of the Linnean Society, 147, 399–416.

Snow, B.K. & Snow, D.W. (1988) Birds and berries: a study of an ecological interaction. Poyser, London

Sorensen, A.E. (1981) Interactions between birds and fruit in a temperate woodland. Oecologia (Berlin), 50, 242-249.

Wheelwright, N.T., Haber, W.A., Murray, K.G., Guindon, C. 1984. Tropical fruit-eating birds and their food plants: a survey of a Costa Rican lower montane forest. Biotropica, 16: 173-192.

**Trophic networks** (from Joern, A. (1983) Host Plant Utilization by Grasshoppers (Orthoptera: Acrididae) from a Sandhills Prairie. Journal of Range Management, 36, 793-797.)**:**

Joern, A. (1979). Feeding patterns in grasshoppers (Orthoptera: Acrididae): factors influencing diet specialization. Oecologia, 38, 325-347.

Mulkern, G.B. (1967) Food selection by grasshoppers. Annual Review of Entomology, 12, 59-78.

Mulkern, G. B., Pruess, K.P., Knutson, H., Hagen, A.F., Campbell, J.B. & Lambley, J.D. (1969) Food habits and preferences of grassland grasshoppers of the North Central Great Plains. Bulletin of the North Dakota Agricultural Experiment Station No. 481.

Otte, D. & Joern, A. (1977) On feeding patterns in desert grasshoppers and the evolution of specialized diets. Proceedings of the National Academy of Sciences U. S. A., 128, 89-126.

Pfadt, R. E. & Lavigne, R.J. (1982) Food habits of grasshoppers inhabiting the Pawnee Site. University of Wyoming Agricultural Experiment Station of Scientific Monograph No. 42.

Sheldon, J.D., & Rogers, L.E. (1978) Grasshopper food habits within a shrub-steppe community. Oecologia, 32, 85-92.

Ueckert, D.N. & Hansen, R.M. (1971) Dietary overlap of grasshoppers on sandhill rangeland in northeastern Colorado. Oecologia, 8, 276-295.

**Other trophic network sources:**

Dyer, L. A., Singer, M. S., Lill, J. T., Stireman, J. O., Gentry, G. L., Marquis, R. J., Ricklefs, R. E., Greeney, H. F., Wagner, D. L., Morais, H. C., Diniz, I. R., Kursat, T. A. & Coley, P. D. (2007) Host specificity of Lepidoptera in tropical and temperate forests. Nature, 448, 696-700.

Bergmann, D.J. (1983). Ecology of grasshopper communities of Ozark cedar glades. Doctoral Dissertation thesis, University of Missouri-Columbia, Columbia, Missouri, USA, 115-120.

Smith, F. W. et al. 1974. Grasshoppers (Acrididae) of the Flint Hills Tallgrass Prairie in Kansas. Agricultural Experimental Station. Kansas State University, Manhattan, KS, pp. 72-145.
